# Arachidonic acid and cancer risk: a systematic review of observational studies

**DOI:** 10.1186/1471-2407-12-606

**Published:** 2012-12-19

**Authors:** Mai Sakai, Saki Kakutani, Chika Horikawa, Hisanori Tokuda, Hiroshi Kawashima, Hiroshi Shibata, Hitomi Okubo, Satoshi Sasaki

**Affiliations:** 1 Department of Social and Preventive Epidemiology, School of Public Health, The University of Tokyo, Tokyo, Japan; 2 Quality Assurance Department, Suntory Wellness Limited, Tokyo, Japan; 3 Institute for Health Care Science, Suntory Wellness Limited, Osaka, Japan

## Abstract

**Background:**

An n-6 essential fatty acid, arachidonic acid (ARA) is converted into prostaglandin E_2_, which is involved in tumour extension. However, it is unclear whether dietary ARA intake leads to cancer in humans. We thus systematically evaluated available observational studies on the relationship between ARA exposure and the risk of colorectal, skin, breast, prostate, lung, and stomach cancers.

**Methods:**

We searched the PubMed database for articles published up to May 17, 2010. 126 potentially relevant articles from the initial search and 49,670 bibliographies were scrutinised to identify eligible publications by using predefined inclusion criteria. A comprehensive literature search yielded 52 eligible articles, and their reporting quality and methodological quality was assessed. Information on the strength of the association between ARA exposure and cancer risk, the dose-response relationship, and methodological limitations was collected and evaluated with respect to consistency and study design.

**Results:**

For colorectal, skin, breast, and prostate cancer, 17, 3, 18, and 16 studies, respectively, were identified. We could not obtain eligible reports for lung and stomach cancer. Studies used cohort (n = 4), nested case-control (n = 12), case-control (n = 26), and cross-sectional (n = 12) designs. The number of subjects (n = 15 - 88,795), ARA exposure assessment method (dietary intake or biomarker), cancer diagnosis and patient recruitment procedure (histological diagnosis, cancer registries, or self-reported information) varied among studies. The relationship between ARA exposure and colorectal cancer was inconsistent based on ARA exposure assessment methodology (dietary intake or biomarker). Conversely, there was no strong positive association or dose-response relationship for breast or prostate cancer. There were limited numbers of studies on skin cancer to draw any conclusions from the results.

**Conclusions:**

The available epidemiologic evidence is weak because of the limited number of studies and their methodological limitations, but nonetheless, the results suggest that ARA exposure is not associated with increased breast and prostate cancer risk. Further evidence from well-designed observational studies is required to confirm or refute the association between ARA exposure and risk of cancer.

## Background

Cancer remains an important health problem worldwide. It is estimated that 58.8 million people died of all causes in 2004 [1]. Deaths from cancer represented around one-eighth of these deaths, although many people who died had cancer even though it was not the direct cause of death. By 2030, it is projected that there will be approximately 26 million new cancer cases and 17 million cancer deaths per year [2]. Given these considerations, the prevention of cancer is a major public health issue around the world.

It is well established that dietary and other lifestyle factors play an important role in cancer control. In terms of dietary factors, earlier studies suggested a relationship between fat intake and the risk of several types of cancer. Prospective cohort studies found no association between fat intake and breast cancer, but a randomised trial organised within the Women’s Health Initiative trial suggested a 9% reduction of borderline significance in breast cancer occurrence with decreased fat intake [3-5]. Analysis of the information in the Multiethnic Cohort Study found that intake of different types of fat indicated no association with overall prostate cancer risk or with non-localised or high-grade prostate cancer [6]. A prospective cohort study and a clinical trial failed to find evidence for an association between fat intake and colorectal cancer [7,8]. A dietary intervention study demonstrated that a reduction in fat intake reduces the risk of skin cancer [9,10], but the evidence from observational studies [11,12] has been controversial. Japan is a high-risk area for stomach and lung cancer, but no association with fat intake and these types of cancer has been suggested [2].

Essential fatty acids, namely n-3 and n-6 fatty acids, are involved in many important biological functions [13-16]. They play a structural role in cell membranes, influencing their fluidity and membrane enzyme activities; in addition, some are the precursors of prostaglandins and other lipid mediators. Arachidonic acid (ARA) is an n-6 essential fatty acid and also a major constituent of biomembranes. It is released from membranes by phospholipase A_2_ and converted into various lipid mediators that exert many physiological actions [17-19]. Many studies have shown that lipid mediators derived from ARA, particularly prostaglandin E_2_ (PGE_2_), are associated with various diseases, which is mainly based on the fact that cyclooxygenase (COX) inhibitors are effective against those conditions [20-24]. PGE_2_ is regarded as enhancing tumour extension as well, but it has been suggested that some other ARA mediators inhibit tumour growth [21-25]. In animal models, ARA administration did not affect tumour extension [26,27]. Some observational studies also suggested no relationship between ARA exposure and cancer risk [28,29]. However, there are the inconsistent observational studies that ARA exposure was positively correlated with the risk of colorectal cancer [30,31]. ARA is one of the major polyunsaturated fatty acid, and this inconsistency is not negligible.

No systematic review or meta-analysis has been conducted to evaluate the long-term effects of ARA intake and blood or tissue ARA composition on the risk of colorectal, skin, breast, prostate, lung, and stomach cancers in free-living populations. The objective of this study was to systematically evaluate available observational studies on the relationship between ARA intake and blood or tissue composition of ARA and the risk of these types of cancer.

## Methods

### Search strategy

The PubMed database (http://www.ncbi.nlm.nih.gov/pubmed/) was searched for observational studies on the relationship between dietary or blood ARA levels with cancer risk that were published up to May 17, 2010. To identify target articles effectively, the strategy for the PubMed search was as follows: keywords for outcome and study types were adopted as commonly used terms representing cancer and study design, whereas terms for exposure were selected from specific words that stand for “arachidonic acid” (see Additional file 1). The initial PubMed search yielded 126 potentially relevant articles.

### Study selection

**Figure 1:**
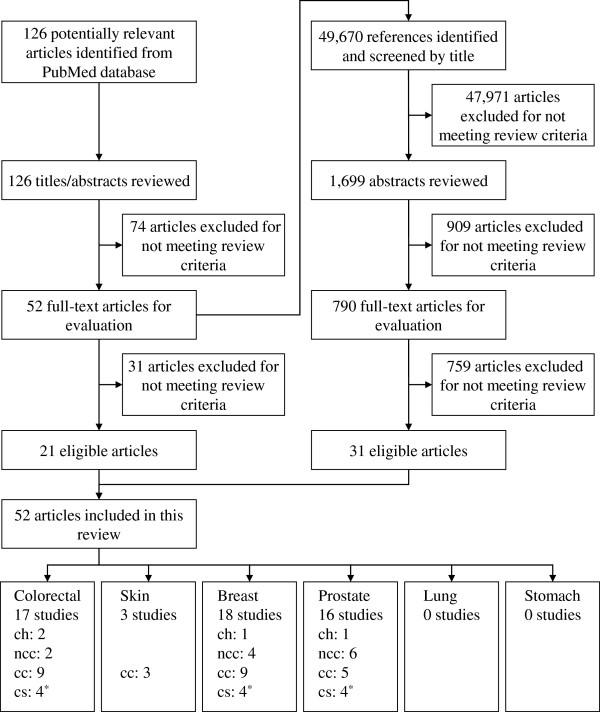
**Flow diagram for literature search and study selection.***CH* Cohort study, *NCC* Nested case-control study, *CC* Case-control study, *CS* Cross-sectional study. *One article that includes data on colorectal, breast and prostate cancer is counted as three studies.

Inclusion criteria were English articles that reported original data on the relationship between ARA exposure (intake or blood level) and target cancer risk in free-living adults. Eligible study designs were cohort, case-control, or cross-sectional studies, and target types of cancer were colorectal, skin, breast, prostate, lung, or stomach cancer. Also included were studies investigating tissue ARA levels and target cancer risk. The study selection process is presented in Figure 1. We omitted reports in which titles or abstracts indicated that: (1) they were not human studies; (2) they were limited to special populations such as people with unusual eating habits; (3) they were intervention studies; or (4) they were not about the target cancers and fatty acids (not fat). We then evaluated the full text of the passed articles. Titles and abstracts of 126 identified publications from the PubMed database were checked and reviewed against the predefined inclusion criteria, and afterward, the full text of 52 articles were similarly assessed for eligibility by three authors (SK, CH, and HT, not independently). The 49,670 bibliographies of these full-text articles were scrutinised to identify additional eligible publications. One article on breast cancer was excluded because an inaccuracy of ARA assessment was clearly reported, although this article met the inclusion criteria described above [32]. Finally, 52 eligible articles were included in this review: 21 and 31 articles were obtained from primary PubMed searches and bibliographies, respectively.

### Quality assessment and data extraction

Quality assessment was conducted based on the reporting quality and methodological quality of each study. The reporting quality shows whether the necessary information for observational studies is well indicated. It is the number of fulfilled items from the Strengthening the Reporting of Observational Studies in Epidemiology Statement (STROBE) checklist and varied 0 to 34 [33]. The reporting quality of included observational studies was assessed individually by two reviewers (CH and HT) and then confirmed by another two authors (SK and MS). The methodological quality, a level of suitability of methods used in a study, was assessed by two authors (SK and MS) qualitatively from the following methodological aspects used in the article: subject selection, ARA exposure assessment, diagnosis or recruitment procedure of cancer patients, methods for controlling confounders, and statistical analysis.

For each eligible article, the following information was tabulated: authors and year of publication, study settings and design, subject characteristics (such as age, sex, and number), matching strategy (if applicable), ARA exposure assessment used (as well as information about validity or precision), outcome assessment, adjusted confounders, reporting quality score from the STROBE checklist, and main findings from the fully adjusted model. Case-control studies were classified into two groups based on whether they reported temporal study settings information between exposure and outcome assessment: “case-control study (temporal relationship among exposure and outcome is demonstrated)” was defined as articles in which ARA exposure preceded the occurrence of cancer, whereas “case-control study (temporal relationship among exposure and outcome is unclear)” did not describe sufficient temporal information about exposure and outcome assessment.

Our qualitative definition of the study quality was as below: the reporting quality score under 13 or the insufficient temporal information, low; the other studies were qualitatively divided into high/medium/low according to their strength and weakness. A meta-analysis was not conducted because of the heterogeneity among studies, particularly subject characteristics and exposure/outcome assessment, and the insufficient number of studies with high methodological quality suitable for a meta-analysis. Therefore, qualitative assessment of ARA intake and cancer risk is presented in this review.

## Results

For colorectal, skin, breast, and prostate cancer, 52 eligible articles were selected from potentially related reports and were included in the present systematic review (Figure 1); the number of each was 17, 3, 18, and 16 studies, respectively. In contrast, we could not identify eligible reports for lung and stomach cancer.

### Colorectal cancer

Major characteristics are shown in Table 1[28,30,31,34-47]. Five reports did not provide sufficient information about the methodology of outcome measurement. Some cohort and case-control studies were adjusted for well-known potential confounders, such as family history, body weight and smoking, and specific factors for colorectal cancer, such as body mass index (BMI), physical activity, alcohol drinking and total energy. No confounding factors were adjusted for in eight articles.

**Table 1:** Summary of observational studies on the association between ARA and risk of colorectal cancer

**References**	**Study**	**Subjects**	**Exposure Assessment**	**Colorectal cancer assessment (diagnosis)**	**Adjustment for potential confounders**	Assessment of reporting quality *	**Main findings**		
							**Intergroup comparison**		** P or Ptrend**
**Study design: cohort study**									
**Exposure assessment: dietary intake**									
Murff et al. 2009 [30]	SWHS, China, 1996-2007, prospective cohort design (7-year biennial follow-up, follow-up rate = 96.7%)	73,243 women aged 40-70, no prior history of cancer	SWHS's FFQ, 77 items, previously validated against 24 x 24-HDR	Self-reported physician diagnosis, combined with annual record linkage with the Shanghai Cancer Registry and Shanghai Vital Statistics database	Age at baseline, total energy intake, smoking status, alcohol intake, physical activity, energy-adjusted total red meat consumption, menopausal status, use of HRT, multivitamin, aspirin, total n-3 PUFA intake, n-6 to n-3 PUFA ratio	18	Dietary ARA intake, g/day, quintile, median	RR (95%CI)	Ptrend
							Q1: 0.02	1.00	0.03
							Q2: 0.03	1.20 (0.87-1.64)	
							Q3: 0.05	1.44 (1.05-1.98)	
							Q4: 0.06	1.61 (1.17-2.23)	
							Q5: 0.09	1.39 (0.97-1.99)	
Lin et al. 2004 [28]	WHS, USA, 1993–2003, prospective cohort design nested randomized, double-blind, placebo-controlled 2 × 2 factorial aspirin and vitamin A trial (average 8.7 years follow-up)	37,547 female health professionals aged ≥45, free of heart disease and cancer except NMSC	FFQ, 131 items, validated against 2 x 7-day WR	Self-reported physician diagnosis, reviewed and confirmed medical diagnoses	Age, treatment assignment, BMI, family history of CRC, colorectal polyps, physical activity, smoking status, alcohol intake, use of HRT, total energy intake	15	Dietary ARA intake, %energy, quintile, median	RR (95%CI)	Ptrend
							Q1: 0.04	1.00	0.55
							Q2: 0.06	0.86 (0.57-1.32)	
							Q3: 0.07	0.84 (0.55-1.28)	
							Q4: 0.09	0.73 (0.47-1.14)	
							Q5: 0.12	0.90 (0.59-1.36)	
**Study design: nested case-control study**									
**Exposure assessment: blood ARA level**									
Hall et al. 2007 [34]	PHS, USA, 1982-1995, nested case-control design within a randomized, double-blind, placebo-controlled factorial aspirin and beta-carotene trial (average 5 and 7 years follow-up)	178 CRC patients, 282 controls, male physicians without history of cancer aged 40-84 years at baseline, 1 case matched with 1-2 controls by age, smoking status	Whole blood fatty acids, GC analysis blinded to case-control status at a time, precision indicated	Self-report, combined with review of medical records	None	23	ARA composition%, geometric mean(95%CI) Case:	ARA composition%, geometric mean(95%CI) Control:	P
							9.77(9.57-9.99)	9.93(9.77-10.10)	Not significant
Kojima et al. 2005 [35]	JACC Study, Japan, 1988-1997, nested case-control design (7 years follow-up)	169 primary CRC patients, 481 controls without previous history of cancer, aged 40-79 years at baseline, 1 case matched with 2-3 controls by age, sex, resident area	Serum fatty acids, GC analysis blinded to case-control status, precision not indicated	Population-based cancer registries, supplemented by death certificates	Age at completing final education, family history of CRC, BMI, smoking status, alcohol intake, intake of green leafy vegetables, physical activity	23	ARA composition, weight % of total serum lipids, quartile	OR (95%CI)	P trend
							Men:	Men:	Men:
							Q1: <3.71	1.00	0.99
							Q2: 3.71-4.619	1.24 (0.55-2.78)	
							Q3: 4.62-5.269	0.79 (0.32-1.96)	
							Q4: ≥5.27	1.16 (0.49-2.75)	
							Women:	Women:	Women:
							Q1: <4.20	1.00	0.40
							Q2: 4.20-4.879	0.67 (0.31-1.46)	
							Q3: 4.88-5.634	0.49 (0.22-1.10)	
							Q4: ≥5.635	0.65 (0.30-1.44)	
**Study design: case-control study (temporal relationship among exposure and outcome is demonstrated)**									
**Exposure assessment: dietary intake**									
Theodoratou et al. 2007 [36]	Survey, UK, 1999-2006, case-control design	1,455 primary CRC patients aged 16-79, 1,455 controls (eligibility criteria not shown), matched by age, sex, resident area	Scotish FFQ, 150 items, validated against 4-day WR, (response rate = case 82%, control 97%)	Not shown	Family history of CRC, total energy intake, total fiber intake, alcohol intake, NSAIDs use, smoking status, BMI, physical activity, total fatty acid intake	20	Dietary ARA intake, mg/day, quartile	OR (95%CI)	Ptrend
							Q1: 0-5.82	1.00	0.163
							Q2: 5.83-8.40	1.09 (0.87-1.37)	
							Q3: 8.41-11.34	0.79 (0.63-1.01)	
							Q4:≥11.35	0.93 (0.72-1.19)	
Nkondjock et al. 2003 [31]	Survey, Canada, 1989-1993, case-control design	402 CRC patients aged 35-79, 688 controls, matched by age, language, place of residence	FFQ, 132 items, validated against 7-day Food Record	Histological diagnosis	Age, BMI, family history of CRC, marital status, physical activity	20	Dietary ARA intake, g/day, quartile	OR (95% CI)	Ptrend
							Q1:<0.06	1.00	0.001
							Q2:0.06-0.09	1.24 (0.84-1.84)	
							Q3:0.10-0.14	1.64 (1.12-2.40)	
							Q4:>0.14	2.11 (1.47-3.06)	
Slattery et al. 1997 [37]	Survey, USA, 1991-1994	1993 CRC patients aged 30-79, 2410 controls without history of CRC (population characteristic partially not shown), matched by age, sex, resident state	CARDIA Diet History Questionnaire, validated against 7 x 24-HDR	Cancer registries	Total energy intake, age at selection, BMI, family history of CRC, physical activity, dietary cholesterol, calcium, fiber, NSAIDs use	19	Dietary ARA intake, g/MJ, quintile	OR (95%CI)	Ptrend
							Men:	Men:	Men:
							Q1:<0.17	1.00	Not shown
							Q2:0.17-0.22	1.25 (0.95-1.65)	
							Q3:0.23-0.26	1.08 (0.81-1.44)	
							Q4:0.27-0.33	1.37 (1.03-1.83)	
							Q5:>0.33	1.17 (0.85-1.61)	
							Women:	Women:	Women:
							Q1:<0.039	1.00	Not shown
							Q2:0.039-0.051	0.99 (0.73-1.33)	
							Q3:0.052-0.063	1.15 (0.86-1.55)	
							Q4:0.064-0.077	0.98 (0.72-1.35)	
							Q5:>0.077	0.98 (0.70-1.37)	
**Exposure assessment: blood ARA level**									
Kuriki et al. 2006 [38]	Survey, Japan, 2002-2004, case-control design	74 CRC patients, 221 controls, aged 20-80 without history of cancer or current diseases, 1 case matched with 3 controls by age, sex, season of blood collection	Erythrocyte phospholipids, GC analysis blinded to case-control status, precision indicated	Histological diagnosis	BMI, habitual exercise, alcohol intake, smoking status, green-yellow vegetable intake, family history of CRC	22	ARA composition, mol%, tertile	OR (95% CI)	Ptrend
							T1: <8.625	1.00	<0.05
							T2: 8.625-10.178	0.91 (0.48-1.73)	
							T3: >10.178	0.42 (0.18-0.95)	
**Study design: case-control study (temporal relationship among exposure and outcome is unclear)**									
**Exposure assessment: dietary intake**									
Busstra et al. 2003 [39]	Survey, Netherlands, 1995-1998, case-control design	52 CRC patients, 57 controls, aged under 75 without history of CRC, colon resection, polyposis coli, inflammatory bowel disease, included subjects with familial HNPCC, matching not indicated	FFQ developed for the Dutch cohorts of the EPIC study, 178 items, validated against 12 x 24-HDR	Histological diagnosis	Age, total energy intake, sex, familial background of HNPCC	13	Dietary ARA intake, g/day, tertile	OR (95% CI)	Ptrend
							T1: <0.02	1.0	0.37
							T2: 0.02-0.04	1.3 (0.4-3.9)	
							T3: ≥0.04	0.6 (0.2-1.8)	
**Exposure assessment: blood ARA level**									
Ghadimi et al. 2008 [40]	Survey, Japan, 1997-2003, case-control design	203 CRA patients, 179 controls (negative faecal occult blood test), matching not indicated	Serum fatty acids (fasting blood), GC analysis, precision indicated	Histological diagnosis	Age, BMI, family history of CRA or CRC, history of diabetes, smoking status, alcohol intake, physical activity, season of data collection	18	ARA concentration, mg/dl, quartile	OR (95%CI)	Ptrend
							Men:	Men:	Men:
							Q1:<17.40	1.00	0.104
							Q2:17.40-19.90	0.60 (0.21-1.68)	Women:
							Q3:19.91-22.50	0.58 (0.21-1.60)	0.001
							Q4:>22.50	0.52 (0.19-1.42)	
							Women:	Women:	Women:
							Q1:<18.05	1.00	0.001
							Q2:18.05-20.50	0.49 (0.19-1.24)	
							Q3:20.51-22.38	0.11 (0.28-0.45)	
							Q4:>22.38	0.11 (0.03-0.43)	
Baró et al. 1998 [41]	Survey, Spain	17 CRC patients aged 35-82, 12 controls aged 33-81 with no malignant diseases, matched by age, resident area	Plasma and erythrocyte fatty acids (fasting blood), GC analysis, precision not indicated	Not shown	None	12	Plasma ARA concentration, mg/dl, mean(SEM)	Plasma ARA concentration, mg/dl, mean(SEM)	P
							Case:	Control:	Plasma:
							18.59(1.31)	21.31(1.22)	Not significant
							Erythrocyte ARA composition%, mean(SEM)	Erythrocyte ARA composition%, mean(SEM)	Erythrocyte:
							Case:	Control:	Not significant
							14.61(0.24)	13.50(0.40)	
Neoptolemos et al. 1990 [42]	Survey, UK	32 CRC patients, 42 controls admitted for elective operations for benign without DM, metabolic disorders, blood transfusions, matched by age, sex, admittance period	Erythrocyte phospholipids (fasting blood), GC analysis, precision not indicated	Not shown	None	13	ARA composition%, median(range)	ARA composition%, median(range)	P
							Case:	Control:	
							20.7(12.8-48.9)	18.0(0.0-47.3)	Not significant
Neoptolemos et al. 1988 [43]	Survey, UK	49 CRC patients aged 49-92, 49 controls with benign diaseases aged 48-90, matched by age, sex	Erythrocyte phospholipids (fasting blood), GC analysis, precision not indicated	Not shown	None	12	ARA composition%, median(range)	ARA composition%, median(range)	P
							Case:	Control:	
							21.8 (15.3-28.4)	23.5 (13.8-32.8)	0.043
**Exposure assessment: tissue ARA level**									
Busstra et al. 2003 [39]	Survey, Netherlands, 1995-1998, case-control design	52 CRC patients, 57 controls, aged under 75 without history of CRC, colon resection, polyposis coli, inflammatory bowel disease, included subjects with familial HNPCC, matching not indicated	Buttock adipose tissue fatty acids, GC analysis, precision not indicated	Histological diagnosis	Age, total energy intake, sex, familial background of HNPCC	13	ARA composition mass%, tertile	OR(95%CI)	Ptrend
							T1: <0.35	1.0	0.42
							T2: 0.35-0.45	2.6 (0.7-8.5)	
							T3: ≥0.45	1.7 (0.5-5.8)	
**Study design: cross-sectional study**									
**Exposure assessment: blood ARA level**									
Almendingen et al. 2006 [44]	Survey, Norway	38 FAP patients aged 24-70 (all colectomized), 160 healthy controls aged 21-66	Serum phospholipids (fasting blood), GC analysis, precision indicated	Diagnosis by endoscopy and histology	None	17	ARA composition weight%, mean(SD)	ARA composition weight%, mean(SD)	P
							Case:	Control:	
							10.96(1.85)	7.26(1.51)	≤0.0001
Fernández-Bañares et al. 1996 [45]	Survey, Spain	22 colonic cancer patients, 27 colonic adenoma patients, 12 controls with benign diseases, no significant differences in sex and age	Plasma phospholipids (fasting blood), GC analysis, precision not indicated	Total fibreoptic colonoscopy	None	13	ARA composition%, mean(SEM) Carcinoma:	ARA composition%, mean(SEM) Controls:	P
							9.38(0.37)	10.2(0.32)	Not significant
							Adenoma:		
							9.95(0.49)		
Hietanen et al. 1994 [46]	Survey, UK, cross-sectional design	20 colon cancer patients aged 38-84, controls, matched by age, sex, smoking status	Erythrocyte phospholipids (fasting blood), GC analysis, precision not indicated	Not shown	None	8	ARA concentration, mg/dl, mean(SD)	ARA concentration, mg/dl, mean(SD)	P
							Case:	Control:	
							18.5(0.6)	20.2(0.5)	<0.05
**Exposure assessment: tissue ARA level**									
Fernández-Bañares et al. 1996 [45]	Survey, Spain	15 colonic cancer patients, 21 colonic adenoma patients, 8 controls with benign diseases	Normal colon mucosa fatty acids, GC analysis, precision not indicated	Total fibreoptic colonoscopy	None	13	ARA composition%, mean(SEM) Carcinoma:	ARA composition%, mean (SEM) Controls:	P
							10.9(0.57)	11.4 (0.88)	Not significant
							Adenoma:		
							12.3(0.55)		
Berry et al. 1986 [47]	Survey, Israel, 1982-1985	155 consecutive colonoscopies (53 carcinoma, 34 benign neoplastic polyps, 68 controls)	Buttock adipose tissue fatty acids, GC analysis, precision indicated	Histological diagnosis	None	13	ARA composition%, mean (SD) Carcinoma:	ARA composition%, mean (SD) Controls:	P
							0.54 (0.2)	0.55 (0.2)	Not significant
							Benign neoplastic polyps:		
							0.52 (0.2)		

*24-HDR* 24-h dietary recall, *ARA* Arachidonic acid, *BMI* Body mass index, *CRA* Colorectal adenoma, *CRC* Colorectal cancer, *DM* Diabetes mellitus, *FAP* Familial adenomatous polyposis, *FFQ* Food frequency questionnaire, *GC* Gas chromatography, *HNPCC* Hereditary non-polyposis colorectal cancer, *HRT* Hormone replacement therapy, *JACC* Japan Collaborative Cohort, *NMSC* Nonmelanoma skin cancer, *NSAIDs* Nonsteroidal antiinflammatory drugs, *OR* Odds ratio, *PHS* Physician's health study, *RR* Relative risk, *SWHS* Shanghai Women's Health Study, *UK* United Kingdom, *USA* United States of America, *WHS* Women's Health Study, *WR* Weighed dietary record.

*Result of the critical evaluation carried out using the STROBE tool.

Dietary ARA intake was estimated in two cohort studies and four case-control studies. Median dietary ARA intake ranged widely from 0.008 to 0.15 g/day, or from 0.04% to 0.07% of energy. Two articles reported a significant increase in colorectal cancer risk. Muff et al. indicated that colorectal cancer risk was significantly increased in the third and fourth quintiles of ARA intake, and that the overall trend was significant (P for trend = 0.03). Nkondjock et al. reported significantly increased colorectal cancer risk in the third and fourth quartiles and significance in the overall trend (P for trend = 0.001).

In seven case-control studies and three cross-sectional studies, the exposure was indicated as blood ARA levels. The precision of blood analysis was mentioned in only four reports, and blinded fatty acid measurement was conducted in only three reports. Five articles showed a significant trend of decreasing colorectal cancer risk or a significant difference of blood ARA levels in cancer subjects. Kuriki et al. found that colorectal cancer risk was significantly decreased in the highest tertile of erythrocyte ARA levels, and that the overall trend was significant (P for trend < 0.05). The remaining four reports, Ghadimi et al., Hietanen et al., Neoptolemos et al. (1988), and Almending et al., were a case-control study with little temporal information between exposure and outcome or a cross-sectional study.

One case-control study with little temporal information between exposure and outcome and two cross-sectional studies investigated tissue ARA levels. The precision of tissue analysis was mentioned in only one article, and none reported masking of disease status. Their reporting quality was generally low.

### Skin cancer

Only three articles were included in the present systematic review. Major characteristics are shown in Table 2[48-50]. Their exposure assessment and subjects’ characteristics were too diverse to be compared to each other.

**Table 2:** Summary of observational studies on the association between ARA and risk of skin cancer

**References**	**Study**	**Subjects**	**Exposure assessment**	**Skin cancer assessment (diagnosis)**	**Adjustment for potential confounders**	Assessment of reporting quality *	**Main findings**		
							**Intergroup comparison**		** P or Ptrend**
**Study design: case-control study (temporal relationship among exposure and outcome is unclear)**									
									
**Exposure assessment: dietary intake**									
									
Hakim et al. 2000 [48]	Survey, USA, case-control design	301 nonmetastatic skin SCC patients aged ≥30, 267 population-baseed controls with no prior history of skin cancer, matched by age, sex	24-HDR of 4 days, validated	Histopathologically diagnosed skin SCC selected from Southeastern Arizona Skin Cancer Registry	Age, sex, total energy intake, history of diagnosed actinic keratosis, tanning ability, freckles on arms, use of sunscreen	22	Dietary ARA intake, g/day, tertile	OR (95% CI)	Ptrend
							T1: ≤0.1	1.00	0.16
							T2: 0.11-0.20	0.86 (0.57-1.29)	
							T3: >0.20	0.70 (0.46-1.08)	
									
**Exposure assessment: blood ARA level**									
									
Harris et al. 2005 [49]	Survey, USA, case-control design	336 nonmetastatic skin SCC patients aged ≥30, 321 controls with no prior history of skin cancer, matched by age, sex, race	Erythrocyte fatty acids (fasting blood), GC analysis, precision indicated	Histopathologically diagnosed skin SCC selected from Southeastern Arizona Skin Cancer Registry	Age, sex, lab, tanning ability, freckles on arms, exclusion of 94 controls with history of prior actinic keratosis	25	ARA composition weight%, quartile	OR (95% CI)	Ptrend
							Q1	1.00	Not shown
							Q2	1.61 (0.92-2.80)	
							Q3	1.40 (0.79-2.49)	
							Q4	2.38 (1.37-4.12)	
**Exposure assessment: tissue ARA level**									
Mackie et al. 1987 [50]	Survey, Australia, 1984-1985	100 primary melanoma patients, 100 controls with no history of malignant skin tumor, matched by age, sex, race	Subcutaneous adipose tissue triglyceride, GC analysis blinded to case-control status, precision not indicated	Selected from Sydney Melanoma Unit	None	10	ARA composition%, mean	ARA composition%, mean	P
							Case:	Control:	
							0.41	0.28	<0.001

24-HDR: 24-h dietary recall, *ARA* Arachidonic, *GC* Gas chromatography, *OR* Odds Ratio, *SCC* Squamous cell caricinoma, *USA* United States of America.

*Result of the critical evaluation carried out using the STROBE tool.

### Breast cancer

Major characteristics are shown in Table 3[29,46,51-66]. Five articles did not provide sufficient information about the methodology of outcome measurement. In addition to general confounding factors, specific factors for breast cancer, such as reproductive factors and history of benign breast disease, were considered in some articles; however, no confounding factors were investigated in eight articles.

**Table 3:** Summary of observational studies on the association between ARA and risk of breast cancer

**References**	**Study**	**Subjects**	**Exposure Assessment**	**Breast cancer assessment (diagnosis)**	**Adjustment for potential confounders**	Assessment of reporting quality *	**Main findings**		
							**Intergroup comparison**		** P or Ptrend**
**Study design: cohort study**									
**Exposure assessment: dietary intake**									
Holmes et al. 1999 [51]	NHS, USA, 1976- 1994, prospective cohort design (14 year biennial follow-up, follow-up rate = 95%)	88,795 female nurses aged 30-55, no prior history of cancer other than nonmelanoma skin cancer	Semiquantitative FFQ, 131 items, validated against 2 x 7-day WR	Self-reported physician diagnosis, deaths identified by family member of participants, postal services and National Death Index, supplemented by medical record	Total energy intake, age, energy-adjusted vitamin A intake, alcohol intake, time period, height, parity, age at first birth, weight change, BMI, age at menopause, menopausal status, use of HRT, family history, benign breast disease, age at menarche	19	%energy increment of dietary ARA intake per day 0.03	RR(95% CI)	P
								1.05(1.00-1.10)	Not shown
**Study design: nested case-control study**									
**Exposure assessment: dietary intake**									
Voorrips et al. 2002 [52]	NLCS, Netherlands, 1986-1992 (6.3 years follow-up), case-cohort design	941 breast cancer patients from entire cohort, 1,598 subcohort members (selection criteria not shown), aged 55-69 at baseline, no prior history of cancer other than nonmelanoma skin cancer, matching not indicated	Semiquantitative FFQ, 150 items, validated against 3 x 3-day DR	All regional cancer registries and Dutch national database of pathology reports	Age, history of benign breast disease, maternal breast cancer, breast cancer in one or more sisters, age at menarche, age at menopause, oral contraceptive use, parity, age at first birth, Quetelet index, educational level, alcohol intake, smoking status, total energy intake, total energy-adjusted fat intake	19	Dietary ARA intake, g/day, quintile, median	RR(95%CI)	Ptrend
							Q1: 0.05	1.00	0.93
							Q2: 0.07	0.80(0.59-1.07)	
							Q3: 0.09	0.84(0.63-1.13)	
							Q4: 0.11	0.80(0.59-1.08)	
							Q5: 0.15	0.99(0.73-1.34)	
**Exposure assessment: blood ARA level**									
Saadatian-Elahi et al. 2002 [29]	NYUWHS, USA, 1985-1995 (average 4.3 years follow-up), nested case-control design	197 breast cancer patients, 197 controls (free of cancer), aged 34-65, matched by age, menopausal status, date of blood sampling, number of blood samplings, day of menstrual cycle	Serum phospholipids, GC analysis, precision indicated	Self-reported physician diagnosis, combined with tumor registries, mortality databases and review of clinical and pathological documents	Family history, age at first full-term birth, total cholesterol, history of treatment for benign breast conditions	19	ARA composition%, quartile	OR(95% CI)	P for the overall categorial variable:
							Q1	1.00	0.80
							Q2	0.79(0.43-1.46)	
							Q3	0.99(0.55-1.81)	Ptrend with the score variable
							Q4	0.81(0.45-1.47)	0.66
Pala et al. 2001 [53]	ORDET study, Italy, 1987-1995 (average 5.5 years follow-up)	71 breast cancer patients, 141 controls (free of cancer), 1 case matched with 2 controls by age, menopausal status at recruitment, daylight-saving period at blood sampling, recruitment center and date	Erythrocyte phospholipids (fasting blood), GC analysis blinded to case-control status, precision indicated	Lombardy Cancer Registry	None (BMI, WHR, age at menarche, age at first birth, age at menopause, months of lactation, parity and educational level were investigated)	23	ARA composition%, tertile	OR(95%CI)	Ptrend
							T1: <16.67	1.00	0.42
							T2: ≥16.67-	1.76(0.88-3.53)	
							<17.94	1.40(0.64-3.10)	
							T3: ≥17.94		
									
Chajès et al. 1999 [54]	Three ongoing cohort studies in Sweden, VIP(1986- 1997), northern Sweden component of the WHO MONICA(1986, 1990 and 1994), MSP(1995-1997), nested case-control design	196 breast cancer patients (VIP 103, MONICA 9, MSP 84), 388 controls (VIP 214, MONICA 6, MSP 168), 1 case matched with 2 controls by age, age of blood sample, sampling center	Serum phospholipids (for VIP and MONICA fasting blood, for MSP very little fasting blood), GC analysis, precision indicated	Regional cancer registry, National Cancer Registry, follow-up for vital status (death) or losses to follow-up determined through local and national population registries	Age at menarche, parity, age at first full-term pregnancy, use of hormones, menopausal status	19	ARA composition%, quartile	OR(95%CI)	Ptrend
							Q1	1.00	0.091
							Q2	0.49(0.24-0.99)	
							Q3	0.48(0.22-1.04)	
							Q4	0.51(0.24-1.09)	
**Study design: case-control study (temporal relationship among exposure and outcome is demonstrated)**									
**Exposure assessment: dietary intake**									
Nkondjock et al. 2003 [55]	Survey, Canada, 1989-1993, case-control design	414 primary breast cancer patients aged 35-79, 688 controls (eligibility criteria not shown), population-based, matched by age, language, place of residence	French version FFQ, >200 items, validated against 7-day FD	Histological diagnosis	Age at first full-term pregnancy, smoking status, family history of breast cancer, history of benign breast disease, marital status, number of full-term pregnancies, total energy intake	20	Dietary ARA intake, g/day, quartile	OR(95%CI)	Ptrend
							Q1	1.00	0.723
							Q2	0.65(0.44-0.97)	
							Q3	1.01(0.70-1.53)	
							Q4	0.86(0.58-1.30)	
**Exposure assessment: blood ARA level**									
Vatten et al. 1993 [56]	Janus Serum Bank, Norway, 1973-1991, case-control design	87 breast cancer patients, 235 controls with no prior history of cancer, matched by age, date of blood sampling	Serum phospholipid, GC analysis blinded to case-control state, precision indicated	National cancer registry linked to Janus Serum Bank donor information	None	20	ARA concentration, mg/l, mean(SD) 78(30)	ARA concentration, mg/l, mean(SD) 79(29)	P
									Not significant
**Exposure assessment: tissue ARA level**									
London et al. 1993 [57]	Survey, USA, 1986-1988, case-control design	Postmenopausal women, 380 breast cancer patients, 573 controls with breast abnormality (free of breast cancer), matching not indicated	Buttock adipose tissue fatty acids, GC analysis, precision indicated	Physician diagnosis (detail not shown)	Age, alcohol intake, age at first birth, parity, family history of breast cancer, age at menopause, age at menarche, history of benign breast disease, weight	19	ARA composition%, quintile	OR(95% CI)	Ptrend
							Q1	1.0	0.60
							Q2	0.8(0.5-1.2)	
							Q3	0.9(0.6-1.5)	
							Q4	1.0(0.6-1.6)	
							Q5	1.0(0.6-1.6)	
**Study design: case-control study (temporal relationship among exposure and outcome is unclear)**									
**Exposure assessment: dietary intake**									
Zhu et al. 1995 [58]	Survey, Finland, 1990-1992	17 premenopausal, 32 postmenopausal primary breast cancer patients, 34 premenopausal, 16 postmenopausal controls with benigh breast disease (eligibility criteria not shown), matching not indicated	Semiquantitative FFQ, 110 items, validated against 14-day DR	Histological diagnosis	Age, total energy intake	13	Dietary ARA intake, mg/day, mean(SD)	Dietary ARA intake, mg/day, mean(SD)	P
							Premenopausal case:	Premenopausal control:	Premenopausal:
							58(27)	163(323)	Not significant
							Postmenopausal case:	Postmenopausal control:	Postmenopausal:
							90(191)	62(26)	Not significant
**Exposure assessment: blood ARA level**									
Aro et al. 2000 [59]	Kuopio Breast Cancer Study, Finland, 1992-1995, case-control design	195 primary breast cancer patients aged 25-75, 208 controls drawn randomly from the National Population Register, matched by age, long-term area of residence	Serum fatty acids (fasting blood), GC analysis, precision indicated	Histological diagnosis	Age, area, age at menarche, age at first full-term pregnancy, use of oral contraceptives, use of HRT, family history of breast cancer, history of benign breast disease, educational level, alcohol intake, smoking status, physical activity, WHR, BMI	15	ARA composition%, quintile, median	OR(95% CI)	Ptrend
							Postmenopausal:	Postmenopausal:	Postmenopausal:
							Q1: 3.84	1.0	Signifncant
							Q2: 4.89	1.1(0.4-2.8)	
							Q3: 5.46	2.0(0.8-4.8)	
							Q4: 6.04	2.4(1.0-5.9)	
							Q5: 7.15	3.1(1.3-7.8)	
							ARA composition%, mean(SD)	ARA composition%, mean(SD)	P
							Premenopausal case:	Premenopausal control:	Premenopausal:
							5.68(1.01)	5.49(1.16)	Not significant
									
Zaridze et al. 1990 [60]	Survey, now-defunct Union of Soviet Socialist Republics, case-control design	25 premenopausal, 21 postmenopausal primary breast cancer patients, 20 premenopausal, 33 postmenopausal neighborhood controls (eligibility criteria not shown), matching not indicated	Erythrocyte phospholipids (fasting blood), GC analysis, precision not indicated	Not shown	None	11	ARA concentration, μg/mg phospholipids, bisectional, (Summer-Autumn/Winter-Spring)	RR(95%CI)	P
							Premenopausal:	Premenopausal:	Premenopausal:
							≤11.70/9.89 vs	0.33(0.08-1.35)	0.122
							>11.70/9.89		
							Postmenopausal:	Postmenopausal:	Postmenopausal:
							≤11.70/9.89 vs	0.23(0.07-0.78)	0.018
							>11.70/9.89		
**Exposure assessment: tissue ARA level**									
Bagga et al. 2002 [61]	Survey, USA, 1995-1996, case-control design	73 breast cancer patients, 73 controls undergoing reduction mammoplasty for mastomegaly, matching not indicated	Breast adipose tissue fatty acids, GC analysis, precision not indicated	Not shown	None	15	ARA concentration, μmol/g total fatty acid, mean(SEM)	ARA concentration, μmol/g total fatty acid, mean(SEM)	P
							Case:	Control:	0.27
							15.03(1.20)	13.13(1.25)	
									
Maillard et al. 2002 [62]	Survey, France, 1992-1996, case-control design	241 patients with non-metastatic invasive breast carcinoma, 88 controls with benign breast diseases, matching not indicated	Breast adipose tissue triglycerides, GC analysis blinded to case-control status, precision indicated	Not shown	Age at diagnosis, height, BMI, menopausal status, BMI-menopausal status interaction	16	ARA composition%, tertile	OR(95% CI)	Ptrend
							T1	1.00	0.32
							T2	0.87(0.41-1.84)	
							T3	0.98(0.42-2.29)	
									
Zhu et al. 1995 [58]	Survey, Finland, 1990-1992	26 premenopausal, 47 postmenopausal primary breast cancer patients, 35 premenopausal, 20 postmenopausal controls with benign breast disease (eligibility criteria not shown), matching not indicated	Breast adipose tissue triglycerides and phospholipids, GC analysis, precision not indicated	Histological diagnosis	Age	13	Triglyceride ARA composition mol%, mean(SD)	Triglyceride ARA composition mol%, mean(SD)	P
							Premenopausal case:	Premenopausal control:	Triglyceride
							0.33(0.18)	0.33(0.27)	Premenopausal:
							Postmenopausal case:	Postmenopausal control:	Not significant
							0.33(0.18)	0.55(0.62)	Postmenopausal:
							Phospholipid ARA composition mol%, mean(SD), Premenopausal case:	Phospholipid ARA composition mol%, mean(SD), Premenopausal control:	<0.01
							9.67(2.56)	9.58(2.17)	Phospholipid
							Postmenopausal case:	Postmenopausal control:	Premenopausal:
							9.64(2.26)	10.95(3.26)	Not significant
									Postmenopausal:
									Not significant
									
Petrek et al. 1994 [63]	Survey, USA, 1987-1989, case-control design	154 invasive breast cancer patients, 125 controls at average risk of breast cancer, matching not indicated	Breast adipose tissue fatty acids, GC analysis, precision not indicated	Histological diagnosis	None	7	ARA composition weight%, mean(SD)	ARA composition weight%, mean(SD)	P
							Case:	Control:	Not significant
							0.40(0.15)	0.39(0.16)	
**Study design: cross-sectional study**									
**Exposure assessment: blood ARA level**									
Williams et al. 1993 [64]	Survey, UK	12 malignant breast disease patients, 10 benign breast disease patients, 22 normal controls	Erythrocyte PIs and PCs (fasting blood), GC analysis, precision not indicated	Histological diagnosis	None	8	ARA composition%, only shown as figure:		P
							Erythrocyte PIs: not significant		PCs:
							Erythrocyte PCs: significantly higher in control compared with benign and malignant group		Malignant/Control:
									<0.02
									Benign/Control:
									<0.02
									
Hietanen et al. 1994 [46]	Survey, UK, cross-sectional design	20 breast cancer patients aged 37-85, controls matched by age, sex, smoking status	Erythrocyte phospholipids (fasting blood), GC analysis, precision not indicated	Not shown	None	10	ARA composition%, mean(SD)	ARA composition%, mean(SD)	P
							Case:	Control:	Not significant
							17.5(0.8)	18.5(1.5)	
									
Punnonen et al. 1989 [65]	Survey, Finland	6 breast cancer patients, 9 normal controls	Erythrocyte phospholipids, GC analysis, precision not indicated	Histological diagnosis	None	5	ARA composition%, mean(SEM)	ARA conposition%, mean(SEM)	P
							Case:	Control:	Not significant
							12.1(1.5)	13.3(0.9)	
**Exposure assessment: tissue ARA level**									
Williams et al. 1993 [64]	Survey, UK	12 malignant breast disease patients, 10 benign breast disease patients, 6 normal controls	Breast tissue PIs and PCs, GC analysis, precision not indicated	Histological diagnosis	None	8	ARA composition%, only shown as figure:		P
							Breast tissue PIs: not significant		PCs:
							Breast tissue PCs: significantly higher in control compared with benign and malignant group		Malignant/Control:
									<0.02
									Benign/Control:
									<0.02
Eid et al. 1988 [66]	Survey, Israel	85 sequential patients (37 carcinoma, 27 fibroadenoma, 21 others)	Breast adipose tissue fatty acids, GC analysis, precision indicated	Not shown	None	8	ARA composition%, mean(SD)	ARA composition, mean(SD)	P
							Carcinoma:	Others:	Not significant
							0.62(0.05)	0.46(0.04)	
							Fibroadenoma:		
							0.78(0.18)		

*ARA* Arachidonic acid, *BMI* Body mass index, *DR* Diet record, *FD* Food record, *FFQ* Food frequency questionnaire, *GC* Gas chromatography, *HRT* Hormone replacement therapy, *MONICA* multinational study for Monitoring of Trends and Cardiovascular Disease study, *MSP* Mammary-Screening Project, *NHS* Nurses' Health study, *NLCS* Netherlands Cohort Study on Diet and Cancer, *NYUWHS* New York University Women's Health Study, *OR* Odds ratio, ORDET study: the Hormones and Diet in the Etiology of Breast Cancer Risk study, *PC* Phosphatidyl-choline, *PI* Phosphatidyl-inositol, *RR* Relative risk, *UK* United Kingdom, *USA* United States of America, *VIP* Västerbotten Intervention Project, *WHR* Waist-to-hip ratio, *WR* Weighed dietary record.

*Result of the critical evaluation carried out using the STROBE tool.

Dietary ARA intake was estimated in one cohort study and three case-control studies. These four showed no significant change in breast cancer risk except in the second quartile of ARA intake in the report by Nkondjock et al.

Six case-control studies and three cross-sectional studies investigated blood ARA levels. The precision of blood analysis was reported in only five articles, and blinded fatty acid measurement was conducted in only two articles. Three articles indicated significant differences in breast cancer risk; however, they were a case-control study with little temporal information between exposure and outcome or a cross-sectional study. Aro et al. reported significantly increased breast cancer risk in the highest quintile of serum ARA in post-menopausal women. The reporting quality of the remaining two articles, those by Zaridze et al. and Williams et al., was quite low.

Five case-control studies and two cross-sectional studies examined tissue ARA levels. The precision of tissue analysis was mentioned in only three articles, and only in one report fatty acids measurement was performed in a blinded fashion. A significant change in breast cancer risk or a significant difference in tissue ARA level was not found, except for breast tissue triglyceride ARA levels in a report by Zhu et al. and breast tissue phosphatidylcholine ARA levels in a report by Williams et al.

### Prostate cancer

Major characteristics are shown in Table 4[46,67-81]. Four articles did not provide sufficient information about the methodology of outcome measurement. As well as well-known confounding factors, specific factors for prostate cancer, for instance BMI, physical activity, and total energy, were considered in some articles; however, no confounding factors were adjusted for in seven articles.

**Table 4:** Summary of observational studies on the association between ARA and risk of prostate cancer

**References**	**Study**	**Subjects**	**Exposure Assessment**	**Prostate cancer assessment (diagnosis)**	**Adjustment for potential confounders**	Assessment of reporting quality *	**Main findings**		
							**Intergroup comparison**		** P or Ptrend**
**Study design: cohort study**									
**Exposure assessment: dietary intake**									
Leitzmann et al. 2004 [67]	HPFS, USA, 1986-2000, prospective cohort design (14 years follow-up)	47,866 health professionals aged 40-65, no prior history of cancer	Semiquantitative FFQ, 131 items, validated against 2 x 1-week DR	Self-reported physician diagnosis supplemented by medical record and pathology report	Age, time period, race, family history of prostate cancer, history of type 2 DM and vasectomy, BMI, height, smoking status, physical activity, total energy intake, % of energy from protein intake, monounsaturated fat intake, saturated fat intake and trans unsaturated fat intake, calcium intake, supplemental vitamin E and lycopene	21	Dietary ARA intake, %energy, quintile	RR(95% CI)	Ptrend
							Q1: <0.028	1.00	0.44
							Q2: 0.028-0.035	1.06(0.94-1.19)	
							Q3: 0.036-0.041	1.04(0.92-1.18)	
							Q4: 0.042-0.049	1.02(0.89-1.16)	
							Q5: >0.049	1.08(0.94-1.25)	
**Study design: nested case-control study**									
**Exposure assessment: dietary intake**									
Männistö et al. 2003 [68]	ATBC study, Finland, 1985-1993, nested case-control design (5-8 years follow-up)	198 prostate cancer patients, 198 controls (free of prostate cancer) matched by age, trial supplementation group	Self-administered dietary questionnaire, 276 items, validated against 12 x 2-day DR	Finnish Cancer Registry and Register of Causes of Death	Resident area, educational level, BMI, alcohol intake, smoking period	23	Dietary ARA intake, g/day, median	OR(95%CI)	Ptrend
							Q1: 0.04	1.00	0.23
							Q2: 0.06	0.89(0.52-1.54)	
							Q3: 0.07	1.10(0.64-1.90)	
							Q4: 0.10	1.31(0.77-2.21)	
		
Schuurman et al. 1999 [69]	NLCS, Netherlands, 1986-1992 (6.3 years follow-up), case-cohort design	642 primary prostate cancer patients from entire cohort, 1,525 subcohort members (selection criteria not shown) aged 55-69 at baseline, without prevalent cancer other than skin cancer, matching not indicated	Semiquantitative FFQ, 150 items, validated against 3 x 3-day DR	All regional cancer registries and Dutch national database of pathology reports	Age, family history of prostate carcinoma, socioeconomic status, total energy intake, total energy-adjusted fat intake	23	Dietary ARA intake, g/day, quintile, median	RR(95%CI)	Ptrend
							Q1: 0.06	1.00	0.30
							Q2: 0.09	1.21(0.88-1.66)	
							Q3: 0.11	1.37(1.00-1.87)	
							Q4: 0.13	1.11(0.80-1.54)	
							Q5: 0.17	1.20(0.87-1.66)	
**Exposure assessment: blood ARA level**									
Crowe et al. 2008 [70]	EPIC study, Denmark, Germany, Greece, Italy, Netherlands, Spain, Sweden, UK, 1992-2000, nested case-cohort design	962 prostate cancer patients, 1,061 controls without prevalent cancer other than NMSC, 1 case matched with 1-2 control(s) by study center, age, time of blood sampling, time between blood sampling and last consumption of food or drink	Plasma phospholipids, GC analysis, precision indicated	Regional or national cancer registries or combination of health insurance records, cancer and pathology registries and self-report	BMI, smoking status, alcohol intake, educational level, marital status, physical activity	26	ARA composition mol%, quintile	RR(95%CI)	Ptrend
							Q1: 4.40–7.93	1.00	0.419
							Q2: 7.93–8.89	1.28(0.96-1.70)	
							Q3: 8.90–9.86	1.17(0.88-1.56)	
							Q4: 9.86–10.98	0.81(0.60-1.10)	
							Q5: 10.99–19.14	0.91(0.65-1.25)	
									
Chavarro et al. 2007 [71]	PHS, USA, 1982-1995, nested case-control design within a randomized, double-blind, placebo-controlled factorial aspirin and beta-carotene trial (13 years follow-up)	476 prostate cancer patients, 476 controls, male physicians without history of cancer except NMSC, 1 case matched with 1 control by age, smoking status, with consideration for trial intervention	Whole blood fatty acids, GC analysis blinded to case-control status, precision indicated	Self-report, combined with review of hospital records and pathology reports	Age, smoking status, length of follow-up	22	ARA concentration (%,), quintile, median	OR(95%CI)	Ptrend
							Q1: 7.9	1.00	0.98
							Q2: 9.3	1.22(0.82-1.81)	
							Q3: 10.1	1.05(0.70-1.57)	
							Q4: 10.9	0.98(0.66-1.46)	
							Q5: 12.3	1.09(0.72-1.64)	
									
Männistö et al. 2003 [68]	ATBC study, Finland, 1985-1993, nested case-control design (5-8 years follow-up)	198 prostate cancer patients, 198 controls (free of prostate cancer) matched by age, trial supplementation group	Serum cholesterol ester fatty acids, GC analysis, precision indicated	Finnish Cancer Registry and Register of Causes of Death	Resident area, educational level, BMI, alcohol intake, smoking period	23	ARA composition %, quartile, median	OR(95%CI)	Ptrend
							Q1: 3.96	1.00	0.34
							Q2: 4.55	1.05(0.60-1.84)	
							Q3: 5.09	0.94(0.54-1.64)	
							Q4: 5.89	1.39(0.79-2.44)	
									
Harvei et al. 1997 [72]	Janus serum bank, Norway, 1973-1994, nested case-control design	141 prostate cancer patients, 282 controls (eligibility criteria not shown), 1 case matched with 2 controls by age, date of blood sampling, resident area	Serum phospholipids, GC analysis, blinded to case-control status, precision not indicated	Cancer Registry and Statistics Norway	None	14	ARA concentration mg/l, quartile, upper limit	OR(95%CI)	Ptrend
							Q1: 4.86	1.0	0.6
							Q2: 5.68	1.1(0.6-1.9)	
							Q3: 6.68	1.2(0.7-2.1)	
							Q4: >6.68	0.8(0.4-1.5)	
Gann et al. 1994 [73]	PHS, USA, 1982-1988, nested case-control design within a randomized, double-blind, placebo-controlled factorial aspirin and beta-carotene trial (6 years follow-up)	120 prostate cancer patients, 120 controls, male physicians without history of cancer except NMSC, 1 case matched with 1 control by age, smoking status without regard to trial intervention	Plasma cholesterol ester fatty acids, GC analysis blinded to case-control status, precision indicated	Self-report, combined with review of medical records	None	19	ARA composition of plasma cholesterol estel %, quartile	OR	Ptrend
							Q1	1.00	0.76
							Q2	1.81	
							Q3	1.00	
							Q4	1.36(vs Q1 95% CI: 0.63-2.90)	
**Study design: case-control study (temporal relationship among exposure and outcome is unclear)**									
**Exposure assessment: dietary intake**									
Hodge et al. 2004 [74]	Survey, Australia, 1994-1997, case-control design	858 prostate cancer patients aged <70, 905 controls matched by age	Melbourne FFQ, 121 items, validated against 2 x 4-day WFR	Not shown	Age at selection, study center, calendar year, family history of prostate cancer, country of birth, socioeconomic status	18	Dietary ARA intake, g/day, quintile	OR(95%CI)	Ptrend
							Q1: <0.028	1.0	0.6
							Q2: 0.028-0.036	1.2(0.8-1.6)	
							Q3: 0.037-0.046	1.2(0.8-1.6)	
							Q4: 0.047-0.059	1.0(0.7-1.3)	
							Q5: ≥0.06	1.0(0.7-1.4)	
**Exposure assessment: blood ARA level**									
Ukori et al. 2010 [75]	Survey, USA and Nigeria, case-control design	48 African American and 66 Nigerian prostate cancer patients, 96 African American and 226 Nigerian controls, aged ≥40, without any cancer history other than skin cancer, matching not indicated	Plasma fatty acids (fasting blood), GC analysis, precision not indicated	Abnormal DRE and/or abnormal PSA (>4ng/ml) with histological diagnosis	Age, educational level, family history of prostate cancer, WHR	14	ARA concentration μg/ml, quartile American African:	OR(95%CI)	Ptrend
							Q1 vs Q4	American African:	American African:
							Nigerian:	0.3(0.08-1.11)	
							Q1 vs Q4	Nigerian:	<0.05
								0.75(0.32-1.74)	Nigerian:
									Not significant
Ukori et al. 2009 [76]	Survey, Nigeria, case-control design	66 prostate cancer patients, 226 controls, aged ≥40, matching not indicated (same population as Nigerian participants of Ukori et al. 2010)	Plasma fatty acids (fasting blood), GC analysis, precision not indicated	Abnormal DRE and/or abnormal PSA (>4ng/ml) with histological diagnosis	Age, educational level, family history of prostate cancer, WHR	11	ARA concentration μg/ml, quartile	OR(95%CI)	Ptrend
							Q1	1.00	0.06
							Q2	2.59(0.85-7.86)	
							Q3	1.93(0.73-5.14)	
							Q4	0.75(0.32-1.74)	
									
Newcomer et al. 2001 [77]	Survey, USA, case-control design	67 prostate cancer patients, 156 population-based controls, 1 case matched with about 2 controls by age distribution	Erythrocyte fatty acids, GC analysis blinded to case-control status, precision indicated	Not shown	Age	23	ARA composition weight%, quartile	OR(95%CI)	Ptrend
							Q1: ≤13.25	1.0	0.88
							Q2: 13.26-14.12	1.6(0.7-3.7)	
							Q3: 14.13-14.90	1.6(0.7-3.5)	
							Q4: ≥14.91	0.9(0.4-2.3)	
									
Yang et al. 1999 [78]	Survey, Korea	19 prostate cancer patients, 24 benign prostatic hyperplasia patients, 21 normal controls, matched by age, demographics	Serum fatty acids, GC-MS analysis, precision not indicated	Not shown	None	4	ARA composition%, mean (SD)	ARA composition%, mean(SD)	P
							Cancer:	Normal control:	Not significant
							0.77(0.31)	1.15(0.45)	
							Benign:		
							0.95(0.16)		
**Study design: cross-sectional study**									
**Exposure assessment: blood ARA level**									
Faas et al. 2003 [79]	Survey, USA, 1995-1998	Prostate cancer patients, benign prostate disease patients	Erythrocyte and plasma phospholipids, GC analysis, precision not indicated	Pathology reports	None	10	Erythrocyte ARA composition%, mean(SEM)	Erythrocyte ARA composition%, mean(SEM)	P
							Malignant:	Benign:	Erythrocyte:
							16.33(0.28)	16.68(0.25)	Not significant
							Plasma ARA composition%, mean(SEM)	Plasma ARA composition%, mean(SEM)	Plasma:
							Malignant:	Benign:	Not significant
							12.60(0.27)	13.03(0.29)	
Hietanen et al. 1994 [46]	Survey, UK, cross-sectional design	10 prostate cancer patients aged 64-85, controls, matched by age, sex, smoking status	Erythrocyte phospholipids (fasting blood), GC analysis, precision not indicated	Not shown	None	8	ARA composition%, mean(SD)	ARA composition%, mean(SD)	P
							Case:	Control:	Not significant
							17.8(1.3)	18.6(1.3)	
									
Chaudry et al. 1991 [80]	Survey, UK	20 patients admitted for prostatic surgery (10 malignant, 10 benign)	Plasma phospholipids (fasting blood), GC analysis, precision not indicated	Histological diagnosis	None	6	ARA composition%, median(IQR)	ARA composition%, median(IQR)	P
							Malignant:	Benign:	Not significant
							8.93(1.84)	8.78(2.03)	
**Exposure assessment: tissue ARA level**									
Faas et al. 2003 [79]	Survey, USA, 1995-1998	Prostate cancer patients, benign prostate disease patients	Prostate tissue phospholipids, GC analysis, precision not indicated	Pathology reports	None	10	ARA composition%, mean(SEM)	ARA composition%, mean(SEM)	P
							Malignant:	Benign:	<0.001
							15.20(0.33)	16.99(0.29)	
									
Mamalakis et al. 2002 [81]	Survey, Greece, 1997-1999	36 prostate cancer patients, 35 benign prostate hyperplasia patients	Gluteal adipose tissue and prostate tissue fatty acids, GC analysis, precision not indicated	DRE, serum PSA, transrectal ultrasound, prostate biopsy	None	12	Gluteal adipose tissue ARA composition%, mean(SD)	Gluteal adipose tissue ARA composition%, mean(SD)	P
							Malignant:	Benign:	Gluteal adipose tissue:
							0.28(0.12)	0.25(0.14)	Not significant
							Prostate tissue ARA composition%, mean(SD)	Prostate tissue ARA composition%, mean(SD)	
							Malignant:	Benign:	Prostate tissue:
							5.99(3.65)	10.71(2.69)	<0.001
Chaudry et al. 1991 [80]	Survey, UK	20 patients admitted for prostatic surgery (10 malignant, 10 benign)	Prostate tissue phospholipids, GC analysis, precision not indicated	Histological diagnosis	None	6	ARA composition%, median(IQR)	ARA composition%, median(IQR)	P
							Malignant:	Benign:	
							11.33(4.12)	15.55(2.54)	0.002

*ARA* Arachidonic acid, ATBC Study: Alpha-tocopherol. Beta-carotene cancer prevention study, *BMI* Body mass index, *DM* Diabetes mellitus, *DR* Diet record, *DRE* Digital rectal examination, *EPIC* European prospective investigation into cancer and nutrition, *FFQ* Food frequency questionnaire, *GC* Gas chromatography, *HPFS* Health professionals follow-up study, *IQR* Interquartile range, *NLCS* Netherlands cohort study on diet and cancer, *NMSC* Non-melanoma skin cancer, *OR* Odds ratio, *PHS* Physician's health study, *PSA* Serum level of prostate specific antigen, *RR* Relative risk, *UK* United Kingdom, *USA* United States of America, *USDA* United states Department of Agriculture, *WFR* Weighed food record, *WHR* Waist-to-hip ratio.

*Result of the critical evaluation carried out using the STROBE tool.

One cohort study and three case-control studies examined dietary ARA intake. They showed no significant change in prostate cancer risk according to increased ARA intake.

Blood ARA levels were estimated in nine case-control studies and three cross-sectional studies. The precision of blood analysis was mentioned in only five articles, and masking of disease status was conducted in only four. Ukori et al. (2010) reported that prostate cancer risk of African-Americans decreased in the fourth quartile of blood ARA level, and that the overall trend was significant (P for trend < 0.05). A significant change in prostate cancer risk or a significant difference in blood ARA levels was not found in the other 11 articles.

Three cross-sectional studies examined tissue ARA levels. All of them reported significant decreases of tissue ARA levels in cancer subjects; however, their reporting quality was generally quite low. None of them mentioned the precision of tissue analysis and masking of groups.

## Discussion

In the present review, we systematically reviewed observational studies investigating the association between ARA and cancer of six organs in free-living populations. Fifty-two eligible articles were obtained from our search strategy, and 31 out of the 52 articles were identified from hand searches for references (Figure 1). Thus, reference searching serves an important role in comprehensive literature searches. This pointed out the characteristics of the reporting style of the observational studies for ARA and cancer risk.

Among the 31 eligible articles from reference searches, 22 were not recognised by our PubMed search formula due to keywords related to “exposure”, three were not recognised due to keywords related to “study types”, and six were not recognised due to both. For “exposure” terms, 26 articles could be identified by the addition of the search term “fatty”. The remaining two articles related to the term “exposure” reported fatty acid compositions of tissues only. In the case of “study type” terms, none of the nine articles used a general study design word (i.e., cohort, case-control, or cross-sectional), although the STROBE statement recommends that authors should indicate the study design with a commonly used term in the title or abstract. These reporting characteristics made it difficult to effectively search for observational studies with a focus on individual fatty acids such as ARA. We therefore adopted the search strategy described above.

The findings from articles for colorectal cancer differ depending on the methodology of ARA exposure assessment. A positive dose-response relationship between dietary ARA intake and colorectal cancer was indicated in two reports [30,31], whereas four articles [38,40,43,46] indicated a negative association or significant ARA decrease with blood ARA levels, and no article reported a positive relationship between colorectal cancer risk and tissue ARA level. These inconsistent results seem to indicate that there is little firm evidence that ARA correlates with the risk of cancer.

There were limited numbers of studies on skin cancer, and they varied in the assessment method used for ARA exposure and the target cancer. It is therefore impossible to draw any conclusions from the results.

Among studies for breast and prostate cancer, a strong positive association and a clear dose-response relationship between increased cancer risk and ARA exposure were not observed, although the results were replicated in different settings using different methods. This suggests that ARA exposure is not associated with increased breast and prostate cancer risk.

We suppose that the contradictory findings mentioned above were caused by four main factors. First, methodologies for estimating dietary ARA intake have not been developed sufficiently. Most adults with mixed diets consume approximately 50 to 250 mg of ARA per day from foodstuffs [82-84], whereas some articles on colorectal and prostate cancer have reported lower values [30,36,39,68,74]. Various validated questionnaires were used in articles which assessed dietary ARA intake, but the validation was not conducted for ARA specifically; total fat, saturated fatty acids, monounsaturated fatty acids, polyunsaturated fatty acids, linoleic acid, or eicosapentaenoic acid intake was assessed, but ARA was not. Since the validity of the estimation of dietary ARA intake is sometimes not enough [82], it should be considered whether exposure assessment is conducted with an appropriate method.

Second, assessment of ARA biomarkers such as blood or tissue ARA levels was diverse; assessed blood fractions included erythrocyte, serum, plasma, or total blood. Tissue sampling was conducted from adipose tissue of buttock or malignant target cancer tissue (i.e., colon, skin, breast, and prostate). Individual biomarkers of fatty acids represent intakes for different time periods [85]. Serum or plasma levels of ARA are considered to reflect dietary intake over a few days, whereas erythrocyte and tissue ARA composition serve as more long-term biomarkers. Overall, habitual dietary ARA intake could not be assessed sufficiently in articles that measured ARA composition of serum or plasma, and this might be one of the causes of inconsistent results among eligible articles.

Third, methodologies of participant selection used in the reports could lead to selection bias. A nested case-control study from a physicians’ health study (aspirin and beta-carotene intervention study) did not consider trial intervention through the participant selection procedure [73]. Also, reporting of participant selection was important; among the total of 52 articles, 28 did not sufficiently report the eligibility criteria, and the sources and methods of participant selection, and 31 did not fully describe the numbers of individuals at each stage of the study and reasons for nonparticipation. This makes it difficult to estimate the effect of selection bias, and therefore, the relationship between ARA intake and cancer risk could not be determined.

Fourth, publication bias based on findings of a significant association could exist, especially in breast and prostate cancers. We evaluated publication bias qualitatively, not using any statistical tests. Most of the significant results were found in the studies with low reporting quality. There is a possibility of publication bias. The results of the studies with low reporting quality may tend to be significant by chance, due to the lack of appropriate design. This suggests that publication bias may affect our review result on breast and prostate cancers, but the effect should be small, because we did not give importance to these studies with low reporting quality.

The biological plausibility of the relationship between ARA intake and cancer risk is still being debated. Previous clinical studies with aspirin or nonsteroidal anti-inflammatory drugs (NSAIDs) have suggested that the cyclooxygenase metabolites of ARA may be associated with risk for colorectal, breast, and prostate cancers [21-24]. Many observational studies, however, have failed to find any association between ARA intake, or its level in blood or tissue, and cancer risk. These controversial findings may be explained by the following three reasons. First, ARA levels of blood or tissue may not always represent dietary intake. Garland et al. and Kobayashi et al. reported that correlations between dietary estimates and the ARA contents of adipose tissue or serum phospholipids were very low [82,86]. Second, the increment of blood or tissue ARA levels may not be connected with the amount of ARA metabolites. Our previous study, in which the supplementation of 240 or 720 mg of ARA per day in healthy elderly persons for four weeks was conducted, investigated plasma PGE_2_ and urinary PGE_2_ metabolites [87]. Their concentrations did not differ significantly with regard to ARA supplementation or time points, although plasma ARA compositions increased dose-dependently. Third, ARA metabolites that are produced by pathways other than the cyclooxygenase pathway may decrease cancer risk. LXA_4_ is an anti-inflammatory mediator produced by the lipoxygenase pathway and is regarded to be a suppressor of tumour growth based on anti-angiogenic properties [25,88]. Aspirin or NSAIDs may not only inhibit the production of cyclooxygenase metabolites, but also divert ARA into the lipoxygenase pathway. However, it is unclear whether LXA_4_ contributes to reduced cancer risk in humans.

In the present study, we reviewed all bibliographies of full-text articles for potential inclusion because reference searches serve an important role in comprehensive literature searches. 49,670 articles were listed, and 99.9% of them were not eligible. We considered that this large exclusion resulted from the many articles in which ARA was not described at all; therefore, we tried another reference search from the bibliographies of articles after their exclusion. Fifty full-text articles from the PubMed database and 146 from the reference search mentioned ARA. A total of 13,657 articles were listed in their bibliographies and their number was reduced to a third; however, we could select 30 eligible articles out of 31 articles that were selected from all full-text searching. The one article remaining that was not selected was a report on skin cancer [50]. This might have resulted from the smaller number of articles identified on PubMed for skin cancer (10 reports) than for colorectal, breast, or prostate cancers (48, 31, or 41 reports). This suggests that reference searches from bibliographies of articles including ARA are more efficient when enough articles are identified from the PubMed database.

This systematic review has limitations. First, studies for inclusion could not be selected independently by two or more reviewers. Our inclusion/exclusion criteria were clear and there were few differences which depended on who was in charge; however that may have introduced a potential selection bias. Second, our search was restricted to English publications and articles from the PubMed database. Furthermore, articles that investigated tissue levels of ARA as an exposure assessment could not be identified comprehensively. We did not set the search terms for ARA levels of tissue before the PubMed search, and identified the articles in the reference search. Third, the search term “fatty” or “fatty acid” was not used in the PubMed search. It led to the efficient search but may cause the possibility that the review may not be completed. Fourth, quality assessment of observational studies is difficult because of the heterogeneity of study designs and methods. The reporting quality was quantitatively expressible using the STROBE checklist; in contrast, the methodological quality could not be quantified and was qualitatively estimated by two independent reviewers. This may have seriously influenced the results and conclusions of the present review.

Note that there are insufficient studies to draw any firm conclusions about the relationship between ARA and cancer risk. Further evidence from well-designed observational studies is required.

## Conclusions

In conclusion, we systematically identified articles that investigated the association between dietary ARA intake or its biomarkers and the risk of colorectal, skin, breast, prostate, lung, or stomach cancer, and only a limited number of observational studies were found (17, 3, 18, 16, 0, and 0 studies were found on colorectal, skin, breast, prostate, lung, or stomach cancer, respectively). Furthermore, most studies had one or more critical limitations, such as the obscurity of temporal information about exposure and outcome, the methodology of ARA exposure assessment, and inadequate treatment of potential confounding factors. These studies did not sufficiently demonstrate any relationships between ARA exposure and cancer risk; however, they seem to suggest that ARA exposure was not related to increased breast or prostate cancer risk because strong positive associations and clear dose-response relationships were not observed. Findings concerning the association between ARA exposure and colorectal cancer were inconsistent between studies. Thus, further evidence from well-designed observational studies is required to confirm or refute the association between ARA exposure and cancer risk.

## Competing interests

This study was supported in part by a grant from Suntory Wellness Limited, Japan. MS, SK, CH, HS, HK, and HS are employees of Suntory Wellness Limited. HO has no competing interests. SS has consultancy relationships with Suntory Wellness Limited.

## Authors' contributions

SK conducted database searches. SK, CH, and HT made decisions on the inclusion and exclusion of the articles. MS and SK conducted quality and bias assessments, contributed to interpretation of findings, wrote the manuscript, and incorporated changes suggested by others. HK, HS, HO, and SS helped to interpret the findings and refined the manuscript. All authors have read and approved the final manuscript

## Pre-publication history

The pre-publication history for this paper can be accessed here:

http://www.biomedcentral.com/1471-2407/12/606/prepub

## Supplementary Material

Additional file 1 PubMed search terms and strategies. Click here for file
