# Supplementary material for: Arachidonic acid and cancer risk: a systematic review of observational studies
Source: BMC Cancer. 2012 Dec 19;12:606. doi: 10.1186/1471-2407-12-606 (PMC3574856; doi:10.1186/1471-2407-12-606)
Supplement: Additional file 1 — PubMed search terms and strategies. [file 1471-2407-12-606-S1.doc]

Appendix: PubMed search terms and strategies

A. Search terms for exposure, outcome, and study types

| Number | Items | Terms |
| --- | --- | --- |
| Exposure | | |
| #1 | Intake | Humans[mesh] AND (arachidonic OR arachidonate OR arachidonates OR "20:4" OR "C20:4" OR eicosatetraenoic) AND ((dietary OR diet OR diets) OR (intake OR intakes OR consumption)) |
| #2 | Biomarker | Humans[mesh] AND (arachidonic OR arachidonate OR arachidonates OR “20:4” OR “C20:4” OR eicosatetraenoic) AND (blood OR serum OR plasma OR erythrocyte OR erythrocites OR “red cell” OR “red cells”) AND ((composition OR compositions OR profile OR profiles OR ratio OR ratios OR status OR statuses) OR (concentration OR concentrations OR level OR levels)) |
| Outcome | | |
| #3 | Colorectal cancer | colorectal neoplasms[mesh] OR colonic neoplasms[mesh] OR intestinal polyps[mesh] OR adenomatous polyps[mesh] OR ((benign*[tiab] OR malign*[tiab] OR neoplasm*[tiab] OR carcinoma*[tiab] OR cancer*[tiab] OR tumor[tiab] OR tumors[tiab] OR tumoral*[tiab] OR tumori*[tiab] OR tumorlet*[tiab] OR tumour*[tiab] OR polyp[tiab] OR polyps[tiab] OR polypu*[tiab] OR polypi[tiab] OR adenom*[tiab]) AND (colon[tiab] OR colonic[tiab] OR rectum[tiab] OR rectal[tiab] OR colorectum[tiab] OR colorectal[tiab] OR large bowel[tiab] OR large intestine*[tiab] OR gut*[tiab])) |
| #4 | Skin cancer | skin neoplasms[mesh] OR melanoma[mesh] OR ((benign*[tiab] OR malign*[tiab] OR neoplasm*[tiab] OR carcinoma*[tiab] OR cancer*[tiab] OR tumor[tiab] OR tumors[tiab] OR tumoral*[tiab] OR tumori*[tiab] OR tumorlet*[tiab] OR tumour*[tiab] OR polyp[tiab] OR polyps[tiab] OR polypu*[tiab] OR polypi[tiab] OR adenom*[tiab]) AND (skin*[tiab] OR cutis[tiab] OR cutaneous*[tiab])) OR (melanoma[tiab] OR melanocarcinoma[tiab] OR melanocytoma[tiab]) |
| #5 | Breast cancer | breast neoplasms[mesh] OR ((benign*[tiab] OR malign*[tiab] OR neoplasm*[tiab] OR carcinoma*[tiab] OR cancer*[tiab] OR tumor[tiab] OR tumors[tiab] OR tumoral*[tiab] OR tumori*[tiab] OR tumorlet*[tiab] OR tumour*[tiab] OR polyp[tiab] OR polyps[tiab] OR polypu*[tiab] OR polypi[tiab] OR adenom*[tiab]) AND (breast[tiab] OR mammary[tiab])) |
| #6 | Prostate cancer | prostatic neoplasms[mesh] OR ((benign*[tiab] OR malign*[tiab] OR neoplasm*[tiab] OR carcinoma*[tiab] OR cancer*[tiab] OR tumor[tiab] OR tumors[tiab] OR tumoral*[tiab] OR tumori*[tiab] OR tumorlet*[tiab] OR tumour*[tiab] OR polyp[tiab] OR polyps[tiab] OR polypu*[tiab] OR polypi[tiab] OR adenom*[tiab]) AND prostat*[tiab]) |
| #7 | Lung cancer | lung neoplasms[mesh] OR ((benign*[tiab] OR malign*[tiab] OR neoplasm*[tiab] OR carcinoma*[tiab] OR cancer*[tiab] OR tumor[tiab] OR tumors[tiab] OR tumoral*[tiab] OR tumori*[tiab] OR tumorlet*[tiab] OR tumour*[tiab] OR polyp[tiab] OR polyps[tiab] OR polypu*[tiab] OR polypi[tiab] OR adenom*[tiab]) AND (lung[tiab] OR pulmon*[tiab] OR chest[tiab])) |
| #8 | Stomach cancer | stomach neoplasms[mesh] OR ((benign*[tiab] OR malign*[tiab] OR neoplasm*[tiab] OR carcinoma*[tiab] OR cancer*[tiab] OR tumor[tiab] OR tumors[tiab] OR tumoral*[tiab] OR tumori*[tiab] OR tumorlet*[tiab] OR tumour*[tiab] OR polyp[tiab] OR polyps[tiab] OR polypu*[tiab] OR polypi[tiab] OR adenom*[tiab]) AND (stomach[tiab] OR gaster[tiab] OR gastri*[tiab] OR gastroc*[tiab] OR gastroi*[tiab])) |
| Study types | | |
| #9 | Study design | (epidemiology OR epidemiologic OR epidemiological) OR (prospective OR cohort) OR ("case control" OR (case AND control) OR "control subjects" OR "control group") OR "cross sectional" |

B. PubMed search strategy for each cancer type

| Cancer site | Term combination |
| --- | --- |
| Colorectal cancer | (#1 OR #2) AND #3 AND #9 |
| Skin cancer | (#1 OR #2) AND #4 AND #9 |
| Breast cancer | (#1 OR #2) AND #5 AND #9 |
| Prostate cancer | (#1 OR #2) AND #6 AND #9 |
| Lung cancer | (#1 OR #2) AND #7 AND #9 |
| Stomach cancer | (#1 OR #2) AND #8 AND #9 |
